# Conflict of interest policies at Belgian medical faculties: Cross-sectional study indicates little oversight

**DOI:** 10.1371/journal.pone.0245736

**Published:** 2021-02-10

**Authors:** Lucas Bechoux, Oriane De Vleeschouwer, Cécile Vanheuverzwijn, Florence Verhegghen, Alizée Detiffe, Fabian Colle, Catherine Fallon, François Thoreau

**Affiliations:** 1 Political Science Department, Spiral Research Center, University of Liège, Liège, Belgium; 2 General Practitioner, *Lhomme et Santé* Medical Center, Saint-Hubert, Belgium; 3 General Practitioner, Triangle Medical Center, Anderlecht, Belgium; 4 General Practitioner, *Cassiopée* Medical Center, Schaerbeek, Belgium; 5 General Practitioner, GRAS, Bruxelles, Belgium; 6 Tropical Medicine/Public Health, Housing First NPO, Brussels, Belgium; University of Toronto, CANADA

## Abstract

**Background:**

Medical students encounter pharmaceutical promotion from the very start of their training. Medical schools have an important role to play in educating medical students regarding the interactions between healthcare professionals (HCPs) and industry and in protecting them from commercial influence and conflict of interest (COI). In 2019, medical student associations in Belgium and abroad called for more preparation in dealing with COI and for a more independent medical training. As little information is available on the situation in our country, we undertook an assessment of conflict of interest policies at Belgium’s medical schools. We relied on a methodology already used in studies from USA, Canada, Australia, France and Germany and adapted it to the Belgian context.

**Methods:**

We identified 10 medical schools in Belgium. We searched the website of each medical school in November 2019 with standardized keywords for COI policies and learning activities on COI in the curriculum. The deans of medicine were invited to participate by sending us information that we could have overlooked during our web-based searches. We also consulted personal contacts within faculties among students and teachers. Based on a list of 15 criteria adapted from North American and French studies, we calculated a total for each faculty of medicine with a maximum score of 30 points.

**Results:**

By December 2019, we had gathered a set of written documents for four faculties of medicine (40%) containing policies with varying degrees of precision and relevance to our survey. We found elements of the curriculum addressing the COI issue for one faculty (10%). In all cases, these policies consisted of “moderate” initiatives with little or no “restrictive” elements. Only one faculty showed interest in our study by providing us with relevant information (10%). Half of the faculty notified us of their refusal to participate in the study (50%) and the other faculties either did not respond or did not provide us with any information (40%). The maximum score obtained was 3 out of 30 points with six faculties scoring 0 (60%).

**Conclusion:**

There is little transparency regarding interactions between medical students and pharmaceutical companies at Belgian medical faculties, which may create COI issues. Initiatives to protect students from pharmaceutical promotion and to train them to manage their future interaction with pharmaceutical companies have a limited scope and are isolated. This is inconsistent with international recommendations from Health Action International, World Health Organization or the American Medical Students’ Association. The Belgian government has legislated in favor of more transparency in the relation between HCPs and pharmaceutical industry. Indeed, it made the disclosure of benefits granted by the industry compulsory and limited their value. Our results show that there is still some way to go to ensure an independent medical training for future Belgian physicians.

## Introduction

Medical drugs are commonly used and a way to improve well-being. In this context, interactions between the pharmaceutical industry, academics and physicians are important: effective partnerships between these different stakeholders have led to major technological innovations and scientific progress.

However, it is important for such interactions to occur within an adequately regulated framework in order to avoid conflict of interest (COI). The misuse of drugs poses significant risks for patients’ health and society in general. Biased information or inappropriate use of medicines may cause more harm than good and put lives at risk [1]. The goals of the pharmaceutical industry and healthcare professionals differ fundamentally in a number of ways. Healthcare professionals (HCPs) need to focus on providing the most suitable treatment, pharmaceutical companies must ensure the sustainability of their business. Here, governments have a role to play in preventing commercial imperatives from overriding the needs of the healthcare system. Neoliberal policies have sometimes left regulators without adequate powers in the face of multinational companies endowed with considerable means to exert influence on practitioners and public authorities (e.g. the Mediator scandal in France in 2010 where the health authorities were unable to protect patients from misuse of the drug due to COI and the influence exerted by the Servier company on the French National Agency for Medicines and Health Products Safety [2]).

Medical students experience interactions with pharmaceutical companies from their first year at the university [3]. An abundant literature has demonstrated the importance of framing conflict of interest and learning how to manage such conflicts early in the training of physicians [4–6]. Several initiatives have emerged around the world to better prepare medical students and protect them from the influence of pharmaceutical companies. Between 2007 and 2016, the American Medical Student Association (AMSA) published an annual ranking of United States medical schools based on a scorecard with criteria assessing their COI policies [7]. This inspired similar initiatives in Australia [8], Canada [9], France [10] and Germany [11]. The United States (USA) has seen significant improvements in COI management at the faculty level, in large part as a result of the AMSA initiatives [12] and the considerable media attention it has generated. In 2007, a majority of medical schools received the worst grade possible (F) on their COI policies. By 2014, however, two thirds of these results evolved to the highest grade (A or B), following the development or strengthening of policies addressing COI [7]. In France, the project of ranking faculties was supported by the National Association of French Medical Students (ANEMF) from its beginning [13]. It triggered a solid movement towards change among academic authorities. The publication of two rankings of French medical schools by *Formindep* (a French association that advocates for more independence in medicine and in medical training) showed very low levels of COI management among the faculties [10]. Following the first one, the National Conferences of Deans of Faculties of Medicine and Deans of Faculties of Dentistry (*Conférences nationales des Doyens de facultés de médecine et des Doyens de facultés d'odontologie*) adopted an ethical charter addressing COI [14]. This shift likely was at least partially due to the intensive media coverage of these rankings in France, indicating growing social concern about pharmaceutical industry influence on medical education.

Throughout 2019, various medical students’ associations adopted positions in favor of a more independent medical training. In their position papers, the International Federation of Medical Students’ Associations (IFMSA) [15] and the European Medical Students’ Association (EMSA) [16] pointed out the influence of the drug industry in medical training and the exposure of students to pharmaceutical marketing. They drew attention to medical students’ lack of preparation for interactions with companies. How to recognize COIs and how to manage them when they occur are subjects of paramount importance to the practice of medicine, yet they are barely touched upon during training. There are many multifaceted forms of influence. The most common promotional techniques are gifts (learning materials, pens, etc.), participation in sponsored educational activities and contacts with pharmaceutical sales representatives [4]. Studies show that students who are exposed early on to pharmaceutical promotion are more likely to develop a positive view of marketing by drug companies [17]. Conversely, those who study in medical schools with strict COI policies are less likely to experience such an influence [18]. Faculty members and the teaching staff may also have ties with the industry through research grants, consultancies, or as speakers or members of advisory committees for companies [19]. These ties may influence their professional medical opinions and introduce bias into the science they teach to the students, compromising the integrity of medical education [20]. Furthermore, teachers are important role models for students, exerting influence on their future prescribing habits and on their future relationship with the pharmaceutical industry [4].

The Belgian Medical Students’ Association (BeMSA) also produced a policy document in 2020 [21] highlighting the need for more independent medical training and a more stringent monitoring of COI in the medical schools and in medicine in general. BeMSA, along with EMSA and IFMSA, published calls to action with recommendations to the medical faculties, teaching hospitals and public authorities to act proactively to reduce potential harm from conflict of interest in medicine.

The Belgian government has introduced legislation to support more transparency in industry promotional practices. Firstly, since January 1, 2007 the law requires any drug manufacturer wishing to organize a scientific event over several calendar days to introduce ahead of time an application for approval from the MDEON ethics platform. HCPs who want to participate in these events are also required to apply for approval [22]. The MDEON platform is a self-regulatory body for the medical sector made up of professional and industry associations. The approvals it grants to organizers and sponsors of medical scientific events are intended to certify that the events comply with the legislation and the MDEON code of ethics [23]. Secondly, the ‘Sunshine Act’ requires Belgian and foreign pharmaceutical and medical device companies to document and publish annually in the Belgian transparency registry *betransparent*.*be* all payments, bonuses and benefits they grant, from January 1, 2017, directly or indirectly, to healthcare professionals, healthcare organizations or patient organizations with a practice or headquarters in Belgium [24]. This legal obligation constitutes an important step on the path to independence by making these financial relationships transparent and public, but there is still a long way to go, especially on the issue of COI and influence during the training years.

Indeed, recent studies have shown that this problem is ongoing. In the United States, a study was carried out in 2017 on students enrolling in 14 United States medical schools. Of 911 premedical students who responded to the survey, 646 (71%) had received or seen someone receiving gifts, small meals (snacks) or samples for pharmaceutical marketing purposes prior to registration at the medical school. These contacts with pharmaceutical promotion generally take place during the clinical experiences (such as shadowing experiences) that are required by medical schools for the admission of future students [25]. In 2016, in Japanese medical schools, over 98% of student interns had accepted a small gift, a notepad, a brochure or a meal from the pharmaceutical industry; 80% of them had participated in a sponsored seminar. Interactions with pharmaceutical companies were found to be significantly more frequent for interns than for students at the start of training [26].

The majority of French medical students (85%) do not feel adequately trained on the issue of conflict of interest [27]. In Germany, most students (90%) say that the question of interactions with pharmaceutical companies is never addressed in the curriculum. More than half of them (65%) do not feel prepared for such interactions and 60% are interested in receiving further training on this subject [28]. Two out of three students in Norway consider themselves incompetent to manage interactions with industry and almost all students think that activities should be organized to prepare them for these interactions [29]. As Austad *et al*. pointed out, education on conflict of interest is not enough [4]. It is important to operate institutional reforms by integrating firm rules to restrict the influence of pharmaceutical companies in medical education. Interventions to reduce contacts between students and industry and to remove gifts provide the groundwork for “healthy skepticism” towards drug promotion and support the practice of evidence-based medicine [18,30].

Inspired by the studies carried out abroad and the encouraging impact it had on the learning environment of medical students, we aimed to assess the institutional COI policies in place in Belgian medical schools. Our team consisted of physicians from the Health Research and Action Group (GRAS) and a social sciences researcher from Liège University. GRAS is a Beglian non-profit association that advocates for more independent medicine [31]. We relied on a list of pre-defined criteria similar to those used in the previous studies [7,9–11] and followed a similar grading system for each criterion to reflect the strength of implemented policies. We also examined whether or not educational activities addressed COI and whether education on management of interactions with the industry was integrated into the curriculum of Belgian medical students.

## Methods

We asked the Hospital-Faculty Ethics Committee (*Comité d'éthique hospitalo-facultaire*) of the Liège CHU whether our study required approval from an ethics committee. As our study focused on policies at an institutional level rather than patient data or personal information, and did not include any human experimentation, we were told that such approval was unnecessary. We relied on a solid methodological precedent to carry out our study. As the French ranking, conducted in 2016 [10] by the *Formindep*, was carried out just before we initiated our study, we adapted their methods to the Belgian context. They in turn had relied on the criteria used by the AMSA [32] and the Canadian researchers [9].

### Data collection

First, we identified the different Belgian universities offering training in the field of medicine and having a faculty of medicine. Out of the eleven Belgian universities, we identified ten faculties of medicine: five Dutch-speaking and five French-speaking. Two universities, Université de Mons (UMons) and Université de Namur (UNamur), only offer an undergraduate medical degree. Nevertheless, we decided to include them because of their role in undergraduate medical training in Belgium. (see **S1 File** for the list of Belgian universities included in the study).

Secondly, in December 2018, two researchers independently searched the websites of each faculty to find information or documents referring to possible institutional policies or materials in the curriculum dealing, directly or indirectly, with COI. For our initial search, we used the same French keywords as those of the *Formindep* ranking (translated into Dutch for the Flemish medical schools): « conflits d'intérêts » (conflict of interest/belangenconflicten), « liens d'intérêts » (competing interests/belangenverstrengeling), « industrie pharmaceutique » (pharmaceutical industry/farmaceutische industrie), « laboratoire pharmaceutique » (pharmaceutical firm/farmaceutisch laboratorium), « charte » (charter/charter) and « règlement intérieur » (internal rules/reglement van orde) [33]. Then, as we found no document with this first website search, we decided to repeat the whole procedure in November 2019 with a translation of keywords in English (in addition to Dutch) for websites of the Flemish faculties. When we found a policy, we recorded it with the date of adoption or the policy’s latest date of review.

Thirdly, in May 2019, we contacted the dean of each medical school by email and registered letter to inform them of our study. The letter indicated the purpose of the study, some background information and explained the documentation we needed. We mentioned that our search of medical school websites had not produced any relevant information on COI policies. We asked whether there were publicly accessible policies addressing the management of COI that we could have missed and, if so, how we could obtain a copy. If such policies were being developed, we also asked when they would be finalized. This allowed the deans and the faculty authorities to provide us with details of any relevant documents or policies. As we were only interested in documents and policies made public by the faculties, we informed the deans that the information would not be kept confidential and that our report would name individual universities. (see **S3** & **S4 Files** for the template of the letter sent to the deans in French and Dutch languages).

In order to enable them to respond, we emailed a series of reminders to the deans of the faculties from which we had not received a response: first reminder August 31, 2019; second, September 30, 2019; and third, November 4, 2019. On December 3, 2019, we sent the deans our intermediate results, in order to enable them to correct these results or to provide any other information that we had not found. A final reminder was sent on December 18, 2019.

Although training hospitals and internships are also important for medical training, we chose to focus our study only on medical faculties. In addition to policies of medical faculties, we also included any university-wide COI policies as such policies also cover the activities of the university’s medical faculty. In order to complete our data collection, we also obtained information through personal contacts with actors in the field inside the universities, such as lecturers or students.

### Scoring criteria

As discussed above, the scoring system is based on criteria used in previous studies in France: Scheffer *et al*. [10] and Canada: Shnier *et al*. [9], adapted to the Belgian context. We retained 12 out of the 14 criteria initially used by Scheffer *et al*. and added three. Since French law prohibits drug samples, the French study had excluded the Canadian study criterion on free samples. Belgian law allows samples [34] as well as premiums and benefits under certain conditions [35]; therefore we included the sample criterion. Much medical training during internships occurs in teaching hospitals. We therefore included a criterion, also used by AMSA and *Formindep*, assessing whether medical faculties promoted the adoption of COI policies in affiliated internship sites. For certain criteria, we provided details—which appear in the MDEON code of ethics [35]—related to provisions in Belgian law. Our list of criteria covered these topics (see **S2 File** for the details about criteria used in the study**)**:

1. Gifts and samples
2. Meals
3. Consulting relationships and advisory role for companies
4. Industry-funded speaking relationships/lecturer services
5. On-site industry-sponsored education activities
6. Compensation for travel or attendance at off-site lectures & meetings
7. Ghostwriting
8. Pharmaceutical sales representatives
9. COI restriction policies
10. Medical school curriculum and learning activities addressing COI
11. Pharmaceutical industry funding of the medical school
12. Medical school activities to promote COI policies in affiliated internship places
13. Use of international Nonproprietary Name (INN)
14. Transparency of funding
15. Implementation and sanctions

As the United States, Canadian and French studies did, we chose to integrate a final criterion of "Implementation and sanctions " in order to take into account the existence of a body responsible for monitoring the rules and policies set out for each of the different criteria, along with the possible existence of sanctions in the event of non-compliance. Similar to the French study, we only assessed the existence of sanctions; we did not verify whether the measures had been violated or the severity of sanctions [10].

Each criterion has a standardized rating scale graduated from 0 to 2. The score of 0 corresponds to the absence of a policy or to a permissive policy; the rating of 1 corresponds to a limited policy and the rating of 2 corresponds to a strong policy. Once we had gathered all available information through the two searches on the faculties' websites, contacts with the deans and personal contacts with students and teachers, we met on November 16, 2019. Two people independently assigned scores to the medical schools for each criterion. Any disagreements were resolved through discussions. Finally, we added the scores obtained to each of the 15 criteria for each faculty, with each criterion assigned equal weight, and arrived at a final score between 0 and 30 points. We then gave each faculty a grade: “A” = 26 points or more; “B” = 22 to 26; “C” = 17 to 22; “D” = 17 points or less and "I" in cases where we could find no information.

## Results

We identified a total of ten Belgian faculties of medicine. All medical schools have a website.

### Web-based searches

Through our web-based searches we found policies for four faculties of medicine: Katholieke Universiteit Leuven (KU Leuven), Université Catholique de Louvain (UCLouvain), Universiteit Gent (UGent) (the most elaborated written policy) and Universiteit Hasselt (UHasselt). These are described in **Table 1.**

**Table 1:** Documents obtained through web-based searches.

	KU Leuven	UCLouvain	UGent	UHasselt
Document name	*Policy and Guidelines for Authorship of Scientific Publications [36]*	*Mission, vision and values [37]*	*Code of conduct: relationship between doctors and industry [38]*	*Integrity charter [39]*
Date	2015	2019	2006	2018
Criteria addressed	Ghostwriting	Learning activities addressing COI in the curriculum; use of INN	Industry-funded lecturer services; ghostwriting; pharmaceutical industry funding of the medical school	COI restriction policies
Policy strength	Moderate policy (not only applicable to medical school)	Moderate policies	Moderate policies	Moderate policy (not only applicable to medical school)

### Mail and email contacts with the Dean’s Offices of the faculties of medicine

We obtained a fairly high response rate of 70% (7 out of 10 faculties) from the Dean’s Offices following the first reminder.

The dean of medicine at Universiteit Antwerpen (UAntwerpen) informed us of the decision by the College of the Flemish deans of medicine to not respond to our request. This meant that half of the deans we contacted explicitly refused to take part in the study.

The new dean of the UCLouvain faculty of medicine, who entered this position at the start of the 2019–2020 academic year, also responded, letting us know that the faculty was very sensitive to this subject and very clearly wanted to participate in our project. We were invited to go to the *Mission*, *Vision and Values* [37] section of the UCLouvain Faculty of Medicine website to find public information. At this point, they were unable to provide us with additional details. The dean added that the question of the independence of medical training and COI would be studied in depth during the year in the context of preparing the AEQES (Belgian agency responsible for assessing the quality of higher education) evaluation.

The dean of the Faculty of Medicine at Université de Liège (ULiège) also replied, asking us for more details on the team carrying out the project. We provided him with information about the GRAS association, but we received no further response.

The other three Dean’s offices did not respond to any of our requests. Therefore, despite a high response rate, contacts with the deans of medical schools provided little information relevant to our study.

In December 2019, we sent our provisional ranking to the deans by email to give them the chance to provide additional information, suggestions or clarifications to our results. The College of the Flemish deans of medicine reiterated their wish not to participate in our study. They told us that they explicitly refused the results of this survey and the ranking that accompanied it. The UCLouvain dean of medicine replied and provided us with relevant information in a section that we had been unable to identify on their website [40]. This page contains information about a moderate restriction on educational activities (including CME) funded by industry. The other four faculties did not respond.

### Data collected through personal contacts with students or teachers

During an interview with a Professor from the Department of General Medicine at ULiège, we discussed the existence of initiatives to prepare students for interactions with industry. Among other courses and activities, the Department aims to integrate in the curriculum the *InfoCritique* [41] learning module for critical reading of information developed by Laval University in Canada.

However, these are only drafts of initiatives or projects with a limited scope that concern only the Department of General Medicine and therefore not all students of the medical school. They therefore cannot be considered as institutional policies and did not contribute to our rankings.

Lastly, a medical student at UCLouvain informed us about the requirement for Master students to participate in the above-mentioned *Infocritiques* module of Laval University. As this constitutes a moderate policy and we had already given one point to UCLouvain concerning elements of the curriculum referring to COI, we decided not to give second point which would have required a strong policy. Detailed analysis of these results are located in **Table 2**. Two universities–UGent and UCLouvain—come first in the ranking, both with a score of 3 points out of 30 (D grade). Then, the faculties of KU Leuven and UHasselt share the second position in the ranking with a score of 1 point (D grade). The six other faculties of medicine (60%)–Université Libre de Bruxelles (ULB), ULiège, UMons, UNamur, UAntwerpen and Vrije Universiteit Brussel (VUB)—for which we found no relevant information totalize a score of 0 point (I grade).

**Table 2:** Conflict of interest policy scores for each Belgian medical school (n = 10).

School	Strength of policy				Enforcement	
	No/permissive	Moderate	Restrictive	Total	Party	Sanction
	Score = 0	Score = 1	Score = 2			
UGent	Gifts and samples	Speaking	-	3	No	No
	Meals	Ghostwriting				
	Consulting	Funding				
	On-site					
	Compensation					
	Sales representatives					
	Restriction					
	Curriculum					
	Promotion					
	INN					
	Transparency					
UCLouvain	Gifts and samples	On-site	-	3	No	No
	Meals	Curriculum				
	Consulting	INN				
	Speaking					
	Compensation					
	Ghostwriting					
	Sales representatives					
	Restriction					
	Funding					
	Promotion					
	Transparency					
KU Leuven	Gifts and samples	Ghostwriting	-	1	No	No
	Meals					
	Consulting					
	Speaking					
	On-site					
	Compensation					
	Sales representatives					
	Restriction					
	Curriculum					
	Funding					
	Promotion					
	INN					
	Transparency					
UHasselt	Gifts and samples	Restriction	-	1	No	No
	Meals					
	Consulting					
	Speaking					
	On-site					
	Compensation					
	Ghostwriting					
	Sales representatives					
	Curriculum					
	Funding					
	Promotion					
	INN					
	Transparency					
ULB	All criteria	-	-	0	No	No
ULiège	All criteria	-	-	0	No	No
UMons	All criteria	-	-	0	No	No
UNamur	All criteria	-	-	0	No	No
Universiteit Antwerpen	All criteria	-	-	0	No	No
VUB	All criteria	-	-	0	No	No

## Discussion

The results of our study reflect inadequate attention among medical schools in Belgium to the issue of independence from pharmaceutical industry or to students’ exposure to COI with industry. Although we found COI policies in 4 faculties (40%), these policies are limited in scope, not clearly restrictive and consist of recommendations rather than prohibitions. None of them cover all of the criteria assessed in this study, and in half of the cases (20%), they are university-wide policies rather than explicitly focusing on medical schools. In addition, information on these policies is difficult to access and requires lengthy searches of the faculty websites. Despite a fairly high response rate from the Deans’ offices, only one faculty (10%) showed interest in our study and agreed to provide us with information (UCLouvain). The other nine faculties (90%) did not respond—or only partially–and five of them notified us of their refusal to participate in our study (50%).

Belgian medical authorities appear to show little interest in this topic and the lack of effort made on this subject by the faculties. We were reported some initiatives within the Department of General Medicine at ULiège to promote independence. However, these are only isolated initiatives carried out by individual professors who are sensitive to the question of independence and do not reflect an institutional decision of the faculty to include this issue in the curriculum. This contrasts with the situation in North America where various official recommendations [42] have pointed out the importance of regulating relations with industry and potential COI during medical education. The position of Belgian medical authorities is even more surprising since, already in 2002, the Royal Academy of Medicine of Belgium (ARMB) drew attention to the problem of relations between doctors and health sector industries [43]. Although there is no research and little information on the extent of influence of the pharmaceutical industry in the training of Belgian students, numerous studies have demonstrated the negative impact of these interactions on prescribing [5,27]. In addition, as mentioned above, the association of Belgian medical students [21] has taken a clear position in favor of greater independence in medical training. These elements should lead the deans and the faculties to take measures in this direction.

On the other hand, problems that university encounters in access to funding cannot be overlooked. The experience of Laval University in Quebec is an example of how hard it is to implement policies to frame interactions between students and companies, in a context of scarce public resources as a result of neoliberal economic policies. In 2009, the university adopted a restrictive policy on COI. In 2015, it had to lower its ambitions due to a lack of sufficient public funding for teaching and research activities within the faculty which led professors to seek funding from pharmaceutical companies, regarding them as partners in the medical training [44]. The COI issue is therefore multifactored and closely linked to the question of the funding of medical training establishments.

As similar studies using the same methodology as ours have been carried out in the United States, Australia, Canada, France and Germany, we are able to compare our results to those in these other countries. Overall, North American medical schools are proactively tackling COI issues in medical education. In 2013, the Canadian study showed the existence of a more or less elaborate form of COI policy in 16 of the 17 existing medical schools, even though 12 of them only obtained a score less than or equal to 50% [9]. In 2014, 133 out of the 161 medical schools in the United States were known to have a COI policy [7]. In Australia, COI policies–again, more or less developed—were identified in 17 faculties out of 20 [8].

In France and Germany, the situation has been more or less similar to what our study revealed. The 2016 French ranking showed that only 2 out of 37 medical schools had policies on COI. Furthermore, these were only informal, unpublished policies rather than clear, written policies. The ranking was repeated in 2018 with criteria modified to reflect the measures the deans had made in their charter. The results showed a clear improvement in the situation with a total of eight faculties obtaining a score greater than or equal to 10 points out of a total of 36 [45]. In Germany, in 2019, only 2 faculties were identified as having policies on COI [11]. However, German medical students are also asking for changes in the way medicine is practiced, starting with bias-free and independent training [46]. It is important to mention here that while we have chosen, in our study, to take into account policies applied at the university level and not only those specific to medical schools, the French and German researchers only considered the latter.

The low scores obtained by the Belgian faculties of medicine compared to the North American faculties could be explained by the fact that in Belgium, apart from the report of the Council of Europe [47], a GRAS article [48] and a note of the Belgian Royal Academy of Medicine [43], there is no official recommendation for more independent medical training. There is a lack of support from public, health and academic authorities in favor of COI policies, unlike in France [49] or in the United States [50]. Additionally, the pharmaceutical industry plays an important role in the Belgian economy; the chemical sector, of which the pharmaceutical industry is an important part, accounts for about 25% of the GDP [51].

We hope that the results of this study will draw the attention of medical school authorities in Belgium to the issue of COI. Belgian faculties of medicine could benefit from greater attention to foreign achievements in terms of position, recommendations and reports. They could also draw inspiration from the rigorous work carried out by AMSA in the aim to reduce as far as possible the influence of the pharmaceutical industry on medical education [52].

In France, a collective of medical students called *La Troupe du RIRE* has produced an awareness booklet on the influence of the pharmaceutical industry during medical training with the aim of making this issue accessible to students [53]. The booklet tackles this sensitive subject with great humor, illustrations and examples while remaining very serious on the substance. After receiving the *Prescrire* prize (that is a prize from the French independent medical journal) in 2015, it has been widely distributed in France; it would be interesting to make it available to Belgian medical students. The University of Bordeaux medical school implemented the FACRIPP (Training in Critical Analysis of Pharmaceutical Promotion) project in 2015. This training aims to provide students with tools enabling them to critically read pharmaceutical promotion [13].

Belgium has also implemented important initiatives in this area. The Belgian Centre of Pharmacotherapeutic Information (CBIP) provides healthcare professionals with independent, neutral and objective information on medical drugs based on scientific evidence. They publish monthly information sheets and maintain an annotated directory of medications updated annually [54]. In collaboration with Farmaka asbl, they publish *Transparency sheets* that are also updated each year. These bulletins synthesize the available data concerning the best drug and non-drug treatments for specified conditions through a review of benefits, the risks and the costs, relying on independent non-commercial information sources [55].

Staff, teachers and students at Belgian medical faculties have many resources and tools available to change their approach to medicine and improve their independence from the pharmaceutical industry. These initiatives could be a source of inspiration for policy development and for educational activities that raise awareness of industry's influence in medicine and the need for independence. As was the case in the United States, the publication of this ranking could have a significant impact on the quality of medical training provided to Belgian students.

## Limitations

Our study has a number of limitations. First, only one in ten faculties we contacted responded and provided us with information. As faculty websites may be incomplete and not contain policy documents, we may have missed information relevant to our study. However, policies that are hard to find and are not formally formulated cannot have the same impact as official, publicly available policies. Moreover, we were unable to verify the results of our internet research for the faculties of KU Leuven, UGent and UHasselt as they did not reply to us to complete or correct our interim results. Only UCLouvain responded and provided additional information.

Internet searches were carried out via the search engines integrated into faculty's websites. These search engines worked differently on different websites. In some cases, they produced a very large volume of results, whether relevant or not. In other cases, they returned almost no results and were limited to predefined terms and expressions. The varying effectiveness of these search engines may have led us to overlook some publicly available documents, policies or educational activities.

Some faculties may be in the process of developing policies that have not yet been implemented. This is the case with the faculty of the UCLouvain, whose dean told us that the question of COI would be studied and closely monitored during the next year. In two cases, we identified policies that do not apply exclusively to medical school, even though COI in medicine requires special attention as it can endanger the lives of patients [56]. Because we only searched the medical school’s websites, we may have overlooked other relevant university-wide policies.

As suggested by Scheffer *et al*. in their conclusions, medical students are most likely to be exposed to pharmaceutical promotion during internships in teaching hospitals [10]. Since this ranking only concerns medical schools, it would be useful to carry out a similar study within Belgian university hospitals. Training of future doctors occurs both at medical faculties and these hospitals. Research to assess how these establishments manage COI with industry would therefore complement the results of our present study. A study in France was carried out to assess COI policies within university teaching hospitals; only 13 out of the 32 major French teaching hospitals had policies covering at least one of the studied criteria [57]. In the United States, AMSA also conducted a survey of COI in university hospitals. They found that out of 204 hospitals, 177 had COI policies by 2014 [58]. This indicates a similar trend to what has been observed for faculty rankings, with higher scores obtained in the United States due to repeated initiatives by AMSA over the past two decades to promote independence of medical education. This positive experience is encouraging.

## Conclusion

In Belgium, addressing the COI issue does not mean starting from zero as there seems to be some reflection underway on the need for independence of medical education. Some faculties have begun to monitor policies on interaction between the pharmaceutical industry and medical students. Preparation of activities to manage this influence is also emerging. However, much remains to be done. Yet, although many initiatives still need to be taken, there is no shortage of clear success stories concerning the management of COI. The situation in Belgium is quite similar to what was observed in France and Germany at the time these countries published their first ranking, with only restricted policies in place in a few faculties. In the United States, where the question of COI in medical education has been tackled for much longer, more progress has been made, most likely because of the importance of the initiative by medical students (AMSA). Official organizations throughout the world as well as medical students’ associations have called for unbiased medical education and for a cultural change in how medicine is practiced. Policies and initiatives to prevent contacts between students and pharmaceutical industry and situations that could lead to COI have shown a real impact on doctors’ prescribing habits [59]. Medical schools in Belgium thus have a key role to play in protecting their student from undue industry influence and in preparing them for interactions with pharmaceutical companies. They also need to sharpen their critical thinking to develop a practice of medicine most appropriate for patients’ well-being. This study aimed to raise awareness on a topic that will be subject to more and more debate in an increasingly medicalized society.

## Supporting information

S1 File List of the different Belgian universities with a medical school and the internet sites used for web searches. (DOCX)

S2 File Criteria list for the assessment of Belgian medical schools’ COI policies. (DOCX)

S3 File Template of the letter sent to the Dean’s offices (French version). (DOCX)

S4 File Template of the letter sent to the Dean’s offices (Dutch version). (DOCX)
