# Supplementary material for: Conflict of interest policies at Belgian medical faculties: Cross-sectional study indicates little oversight
Source: PLoS One. 2021 Feb 10;16(2):e0245736. doi: 10.1371/journal.pone.0245736 (PMC7875358; doi:10.1371/journal.pone.0245736)
Supplement: S1 File — (DOCX) [file pone.0245736.s001.docx]

**S1 File. List of the different Belgian universities with a medical school and the internet sites used for the web searches**

- **Universiteit Antwerpen** https://www.uantwerpen.be/nl/overuantwerpen/faculteiten/geneeskunde-gezondheidswetenschappen/
- **ULB** https://medecine.ulb.be/
- **VUB** https://gf.vub.ac.be/
- **UGent** https://www.ugent.be/ge/nl
- **UHasselt** https://www.uhasselt.be/geneeskunde
- **KU Leuven** https://med.kuleuven.be/nl
- **ULiège** https://www.facmed.uliege.be/cms/c_3211623/fr/faculte-de-medecine
- **UCLouvain** https://uclouvain.be/fr/facultes/mede/med
- **UMons** https://web.umons.ac.be/fmp/fr/
- **UNamur** https://www.unamur.be/medecine/etudes-medecine
