# Supplementary material for: Conflict of interest policies at Belgian medical faculties: Cross-sectional study indicates little oversight
Source: PLoS One. 2021 Feb 10;16(2):e0245736. doi: 10.1371/journal.pone.0245736 (PMC7875358; doi:10.1371/journal.pone.0245736)
Supplement: S2 File — (DOCX) [file pone.0245736.s002.docx]

**S2 File. Criteria list for the assessment of Belgian medical schools’ COI policies**

**Graduation scale:**

**2**: restrictive policy

**1**: moderate policy

**0**: permissive policy or lack of policy

1. **Gifts and samples**

Belgian legal framework: premiums and benefits of negligible value (maximum € 50 per gift and € 125 per year) which relate to the practice of the medical art are authorized.

**2**: All industry-sponsored gifts are prohibited, regardless of the nature or value of the gift.

**1**: Gifts are permitted for a value less than or equal to € 50 per year and limited to gifts of educational value.

**0**: Policy limited to legal framework or no policy.

1. **Meals**

Belgian legal framework: a meal can be offered by industry as part of a scientific event, for a maximum of € 40 for a lunch (drinks included) and € 80 for a dinner (drinks included).

**2**: No meal allowed, whatever its nature and value.

**1**: Meals allowed only if:

- The value of the meal is less than or equal to € 20;
- As part of accredited CME.

**0**: Meals allowed with a value greater than € 20 or no restriction specified.

1. **Consulting relationships and advisory role for companies**

**2**: Consulting relationships and advisory role for companies are permitted but they must meet all of the following criteria:

- Prior institutional review or approval;
- Description in a formal contract;
- Remuneration for services proportional to the work provided;

A register must allow the general public to consult these relationships.

**1**: Consulting relationships and advisory role for companies are permitted but they must meet one of the criteria above-mentioned.

A register must allow the general public to consult these relationships.

**0**: No policy addressing consulting relationships and advisory role for companies.

1. **Industry-funded speaking relationships/lecturer services**

**2**: Academic staff are prohibited from making an industry-sponsored promotional speech or being in an industry-funded speaker's bureau. The university's policy on industry sponsored conferences must meet the following six criteria:

- The speech is not promotional in nature but purely educational;
- Industry has no role in shaping and approving the content of the presentation;
- Long term agreements with industry as a speaker are not permitted;
- Compensation and reimbursement are limited by university policy;
- The requirement to ensure the scientific integrity of the information presented should be mentioned in a policy.
- The details of this policy should be accessible to the general public as well as the directory of conferences already sponsored by the industry.

**1**: Industry-sponsored speaking relationships are regulated but with less stringent limits on content control, financial compensation, etc.

**0**: No limitation on industry sponsored speech or no policy addressing that point.

1. **On-site industry-sponsored education activities**

Belgian legal framework: the financing of an educational activity requires an application for an MDEON visa, which controls compliance with the code of ethics, namely:

- The scientific program constitutes the bulk of the event;
- Speakers, given their function, are supposed to be objective and scientific. There must be a link between the topics covered and the practice of the profession

**2**: Industry is not permitted to provide direct or indirect financial support for educational activities, including continuing medical education (CME), either directly or through a subsidiary agency.

**1**: Industry is permitted to provide direct financial support for educational activities: if the activity is too expensive for physicians without external funding, if the training is accredited and if at least two of the following criteria are met to prevent promotional content:

- The presence of more than one sponsor at the event;
- Lack of financial profit by the organizing department (funds are used only for the educational program);
- Participants must contribute part of the cost of the training;
- The training activity must be managed by a CME program;
- Another rule to reduce the influence of industry

**0**: Financial support from industry is accepted with the sole condition that the training is accredited by the INAMI or no policy addressing that point.

1. **Compensation for travel or attendance at off-site lectures & meetings**

Belgian legal framework: requires an MDEON visa which verifies:

- That the stay is not prolonged;
- The absence of leisure activity;
- That the organization of activities abroad is justified by: the presence of a majority of guests not from Belgium, and that, from a logistical point of view, it makes more sense to organize the event in another country, especially in terms of skills and infrastructure present at the venue.

**2**: Academic staff cannot accept payment, gifts, or industry financial support to attend conferences and meetings.

**1**: Compensation can only be accepted if it is subject to institutional and MDEON approval or if industry is not allowed to select recipients.

**0**: Policy that would not substantially limit attendance at industry funded events and meetings or no policy addressing that point.

1. **Ghostwriting**

**2**: Ghostwriting is not allowed.

**1**: The practice of ghostwriting is discouraged but not prohibited.

**0**: No policy addressing that point.

1. **Pharmaceutical sales representatives**

**2**: Industry representatives are not allowed to enter the medical school or meet with faculty and students. (Exceptions may be made for non-commercial purposes such as training on devices or equipment.)

**1**: Pharmaceutical sale representatives are allowed to meet teaching staff but not students, with two limitations: only in care areas without patients and only by appointment. (Same exceptions as mentioned above.)

**0**: Policy that does not significantly restrict access or no policy addressing that point.

1. **COI restriction policies**

**2**: No conflict of interest accepted for members of the academic staff.

**1**: Academic staff should declare past and present financial ties with industry (e.g.: Consulting and conference services, research grants, etc.) to the university, on a publicly accessible website and disclose these relationships to students.

**0**: No policy addressing that point.

1. **Medical school curriculum and learning activities addressing COI**

**2**: Students are trained to acquire a critical mind regarding the existence of conflict of interest with industry, on the basis of the 5 skills presented in the *AMSA Pharmfree curriculum*. With special attention to:

- The influence on the education and curative practices of physicians.
- The influence on how the efficacy and safety of drugs and devices are demonstrated and regulated.

**1**: The program addresses conflict of interest in a more limited way.

**0**: No policy addressing this point (not addressed in the program or elsewhere)

1. **Pharmaceutical industry funding of the medical school**

We mean funding in a broad sense: research, audiences and classrooms, equipment, etc.

**2**: No funding accepted.

**1**: Funding is accepted but limited. With special care that this funding does not interfere with the mission of the medical school to educate and protect its students from pharmaceutical influences.

**0**: No policy addressing that point.

1. **Medical school activities to promote COI policies in affiliated internship places**

**2**: Active and enforceable policy to encourage other training places (especially teaching hospitals) to respect and follow the medical school's policy on independence.

**1**: Active non-binding policy.

**0**: No policy addressing that point.

1. **Use of international Nonproprietary name (INN)**

**2**: Health products are presented in INN on all course and examination materials. This also applies to medical devices.

**1**: INN is used, but partially.

**0**: INN is not or little used.

1. **Transparency of funding**

**2**: The medical school publishes on its website the funding received from pharmaceutical companies and private organizations.

**1**: The medical school makes funding public, but in an incomplete or difficult to access manner.

**0**: The faculty does not make the funding received public on its website.

1. **Implementation and sanctions**

To verify compliance with the rules, the institutions must have set up a monitoring and sanctioning body.

**2**: There is a committee responsible for the general supervision of the rules and penalties are defined in the event of non-compliance.

**1**: There is: either a committee responsible for monitoring the criteria, or penalties for non-compliance.

**0**: No monitoring policy or sanction
