# Supplementary material for: Conflict of interest policies at Belgian medical faculties: Cross-sectional study indicates little oversight
Source: PLoS One. 2021 Feb 10;16(2):e0245736. doi: 10.1371/journal.pone.0245736 (PMC7875358; doi:10.1371/journal.pone.0245736)
Supplement: S3 File — (DOCX) [file pone.0245736.s003.docx]

Bruxelles, le 27 mai 2019

Monsieur le Doyen,


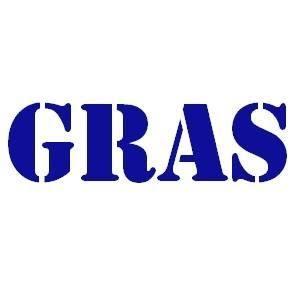


Nous menons une étude ayant pour objet les politiques, accessibles publiquement, des facultés de médecine belges concernant les conflits d'intérêts liés à l'industrie pharmaceutique (entreprises de dispositifs médicaux inclus). Notre recherche concerne les six premières années d'enseignements, jusqu’au concours de spécialisation. Notre méthodologie se base sur les travaux similaires ayant conduit depuis 2007 aux classements des facultés de médecine américaines^^[[1]](#footnote-1)^^, canadiennes^^[[2]](#footnote-2)^^, australiennes^^[[3]](#footnote-3)^^ et françaises^^[[4]](#footnote-4)^^ dont nous avons adaptés les critères d’évaluation au système belge. Nous sommes en lien avec les équipes de recherche canadienne et française. Barbara Mintzes, membre de l’équipe ayant mené l’étude au Canada, nous fait l'honneur de nous apporter son soutien par le biais d’une lettre que vous trouverez ci-jointe. Leur étude a été publiée dans PLoS One, et nous souhaiterions également publier, à terme nos résultats.

**GRAS**

ASBL

Siège social :

Rue de Courcelles, 154

6044 ROUX

www.gras-asbl.be

Les facultés anglo-saxonnes ont développé un ensemble d'initiatives de formation concernant les conflits d'intérêts, et nombre d'entre elles sont citées comme des exemples, que cela soit Stanford aux Etats-Unis (sous l’impulsion de l’AMSA), ou la Western University au Canada. Les résultats positifs, en termes de qualité de prescription et de soins, découlant de ces nouveaux enseignements font déjà l'objet de publications dans les revues médicales de référence^^[[5]](#footnote-5)^^[[6]](#footnote-6)^^. Cette dynamique est d'ailleurs renforcée par la prise de position publique de certains officiels, comme le président de l'Association des facultés de médecine canadiennes, qui souligne la responsabilité des facultés de médecine concernant la formation qu'elles dispensent au sujet des conflits d'intérêts^^[[7]](#footnote-7)^^. Suite à l’étude publiée en janvier 2017 en France par le Formindep, en novembre 2017 la conférence des doyens de médecine et d’odontologie en France a publié une charte^^[[8]](#footnote-8)^^ dans le but de sensibiliser les facultés à l’importance des questions d’indépendance. En Belgique, notre recherche devrait ainsi permettre de se faire une idée précise de la dynamique correspondante, qui n'a fait, à ce jour, l'objet d'aucune étude spécifique.

Les résultats d'une recherche internet sur le site de votre faculté ne nous a pas permis d'identifier un document faisant état de politiques officielles vis-à-vis des conflits d'intérêts. Si vous utilisez d'autres politiques accessibles publiquement en la matière au niveau de la faculté ou de l'université, nous vous serions reconnaissant de bien vouloir nous les signaler, de nous en fournir un exemplaire ou de nous indiquer comment en obtenir un. Si vous avez des politiques en cours d'élaboration ou à l'état de projet, nous vous serions également reconnaissant de nous indiquer quand vous pensez que ces politiques seront finalisées. Vous pouvez retourner votre réponse par courrier (Rue de l'île 4, 5580 Rochefort), ou par mail ([ranking.belgian.faculties@gmail.com](mailto:ranking.belgian.faculties@gmail.com)).

Les politiques de votre établissement seront analysées selon les catégories suivantes : cadeaux et échantillons de médicaments, repas, déclaration publique d'intérêts, contacts avec des délégués commerciaux, organisation d'événements par l'industrie pharmaceutique au sein de la faculté, activités du personnel au sein de comités scientifiques de l'industrie pharmaceutique, enseignements relatifs aux conflits d'intérêts et à l'influence des firmes du médicament et des dispositifs médicaux, utilisation par les enseignants de la Dénomination Commune Internationale (DCI), financements de la faculté liés à l'industrie pharmaceutique, prise en charge des frais de déplacement pour les événements de formation en dehors de l'université, activités de consultant pour le personnel, rédaction anonyme d'articles scientifiques proposée par l'industrie pharmaceutique (ghostwriting), services de conférencier réalisés par le personnel effectués grâce au soutien de l'industrie pharmaceutique, ainsi que la demande de la faculté d'encourager des dispositions similaires auprès des autres lieux de formation (stages en cabinet, à l'hôpital). Enfin, nous souhaiterions savoir si un comité visant à vérifier la mise en place des mesures décidées a été créé, et s’il existe des sanctions en cas de non application des règles par les acteurs concernés. Nous prendrons de nouveau contact avec vous après avoir effectué l'analyse des informations que vous nous aurez communiquées, afin de vous donner la possibilité de confirmer l'exactitude de nos résultats et de notre description de la politique de votre établissement concernant les conflits d'intérêts.

Étant donné que nous ne demandons que les documents publics relatifs à ces politiques, l'information en découlant ne demeurera pas confidentielle, et les facultés de médecine seront identifiées par leur nom. Vous pouvez, bien entendu, refuser de participer ou vous retirer du projet à tout moment. Cependant, toutes les politiques publiques seront retenues même si l'un des doyens contactés se retire.

Pour tout renseignement complémentaire, n'hésitez pas à nous contacter par le biais de l'adresse mail mentionnée précédemment.

Nous vous remercions d'avance pour l'aide que vous pourrez nous apporter dans la réalisation de cette étude importante pour la qualité de la formation des futurs médecins et des soins prodigués aux patients, et pour répondre aux attentes des étudiants, eux-mêmes concernés par cette thématique des conflits d'intérêts. A l’heure des différents scandales qui ont émaillés l’actualité ces dernières années, force est de constater que les thématiques liées à l’indépendance de la profession médicale vis-à-vis de l’industrie pharmaceutique sont plus que jamais dans l’ère du temps.

Dans l'attente de votre réponse, nous vous prions d'agréer nos salutations les plus distinguées.

Les responsables de l’étude :

Elisabeth Bechet

Lucas Bechoux

Fabian Colle

Alizée Detiffe

Oriane De Vleeschouwer

Cécile Vanheuverzwijn

Florence Verhegghen

1. http://www.amsascorecard.org/ [↑](#footnote-ref-1)
2. Shnier A., Lexchin J., Mintzes B., Jutel A., Holloway K., (2013), *Too Few, Too Weak: Conflict of Interest Policies at Canadian Medical Schools,* PLoS ONE [↑](#footnote-ref-2)
3. Mason P., Tattersall M.H.N., (2011), *Conflicts of interest : a review of institutional policy in Australian medical schools*, The Medical Journal of Australia [↑](#footnote-ref-3)
4. Scheffer P, Guy-Coichard C, Outh-Gauer D, Calet-Froissart Z, Boursier M, Mintzes B, et al., (2017), *Conflict of Interest Policies at French Medical Schools: Starting from the Bottom*, PLoS ONE [↑](#footnote-ref-4)
5. Kesselheim A.S., (2013), *Drug company gifts to medical students: the hidden curriculum*, BMJ [↑](#footnote-ref-5)
6. King Marissa, Essick Connor, Bearman Peter, Ross Joseph S., (2013), *Medical school gift restriction policies and physician prescribing of newly marketed psychotropic medications: difference-in-differences analysis*, BMJ [↑](#footnote-ref-6)
7. Busing N., (2011), *Canadian faculties of medicine not in denial*, CMAJ [↑](#footnote-ref-7)
8. http://formindep.fr/charte-ethique-des-conferences-des-doyens-de-medecine-et-dodontologie/ [↑](#footnote-ref-8)
