# Supplementary material for: Conflict of interest policies at Belgian medical faculties: Cross-sectional study indicates little oversight
Source: PLoS One. 2021 Feb 10;16(2):e0245736. doi: 10.1371/journal.pone.0245736 (PMC7875358; doi:10.1371/journal.pone.0245736)
Supplement: S4 File — (DOCX) [file pone.0245736.s004.docx]

Brussel, 27 mei 2019

Geachte decaan,


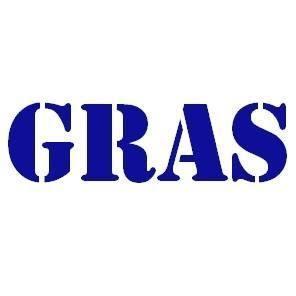


Wij doen onderzoek met als objectief het publiek toegankelijk beleid van de Belgische faculteiten geneeskunde te evalueren m.b.t. belangenconflicten gerelateerd aan de farmaceutische industrie (inclusief bedrijven van medisch materiaal). Ons onderzoek richt zich van de zes (of zeven) eerste studiejaren tot en met de specialisatiejaren. Onze methodologie baseert zich op gelijkaardig onderzoek die sinds 2007 hebben geleid tot de rangschikking van Amerikaanse^^[[1]](#footnote-1)^^, Canadese^^[[2]](#footnote-2)^^, Australische^^[[3]](#footnote-3)^^ en Franse^^[[4]](#footnote-4)^^ geneeskundefaculteiten; de evaluatiecriteria werden aangepast aan het Belgische systeem. We staan ook in contact met de Canadese en Franse onderzoeksgroepen. Barbara Mintzes, lid van de groep die tot de Canadese studie heeft geleid, steunt ons d.m.v. een brief die dit schrijven begeleidt. Hun studie werd gepubliceerd in PloS One en wij zouden ook onze resultaten op termijn willen publiceren.

**GRAS**

ASBL

Siège social :

Rue de Courcelles, 154

6044 ROUX

www.gras-asbl.be

De Angelsaksische faculteiten hebben een geheel aan opleidingsinitiatieven ontwikkeld m.b.t. belangenconflicten, waarvan er vele als voorbeelden worden genoemd, of het nu Stanford in de Verenigde Staten is (onder leiding van AMSA), of Western University in Canada. De positieve resultaten m.b.t. kwaliteit van voorschrijfgedrag en medische zorg voortvloeiend uit nieuwe onderwijsmethodes zijn reeds het onderwerp van publicaties in de gerenommeerde medische tijdschriften^^[[5]](#footnote-5)^^[[6]](#footnote-6)^^. Deze dynamiek wordt versterkt door openbare positionering van gezagsdragers zoals de voorzitter van de vereniging van Canadese geneeskundefaculteiten, die de verantwoordelijkheid van de medische faculteit benadrukt m.b.t. hoeverre het onderwerp van belangenconflicten aan bod komt tijdens de opleiding^^[[7]](#footnote-7)^^. Na publicatie van de studie van Formindep in januari 2017 in Frankrijk werd in november dat jaar een charter^^[[8]](#footnote-8)^^ gepubliceerd tijdens de conferentie van de decanen geneeskunde en tandheelkunde met als doel de faculteiten de sensibiliseren over kwesties m.b.t. onafhankelijkheid. De bedoeling is dat ons onderzoek toelaat om zo in België een idee te vormen van de overeenkomstige dynamiek, welke tot op heden nog geen onderwerp was van een specifieke studie.

Via de website van uw faculteit hebben we geen document kunnen vinden die het officiële beleid m.b.t. belangenconflicten weergeeft. Indien u andere publiek openbare beleidsmaatregelen daaromtrent op niveau van faculteit of universiteit gebruikt zouden wij u bijzonder erkentelijk zijn om ons daarop te duiden, ons daarvan een exemplaar ter beschikking te stellen of ons aan te geven hoe we dit kunnen verkrijgen. Indien beleidsmaatregelen of een project daarrond zouden worden ontwikkeld zouden we u evenzeer erkentelijk zijn ons te duiden wanneer u denkt dat deze zouden zijn afgerond. U kunt uw antwoord per post (Rue de l'île 4, 5580 Rochefort) of per mail (ranking.belgian.faculties@gmail.com) opsturen.

Het beleid van uw organisatie zal volgens volgende categorieën worden geanalyseerd: giften en geneesmiddelenstalen, etentjes, openbare verklaringen van belangen, contacten met commerciële vertegenwoordigers, organisatie van evenementen door de farmaceutische industrie binnen de faculteit, activiteiten van het personeel in wetenschappelijke comités van de farmaceutische industrie, onderwijs m.b.t. belangenconflicten en invloeden van firma’s van geneesmiddelen en medisch materiaal, gebruik van de lesgevers van de International Nonproprietary Name (INN, generieknaam), financiering van de faculteit gerelateerd aan de farmaceutische industrie, bekostiging van verplaatsingskosten voor onderwijsevenementen buiten de universiteit, consultancy-activiteiten voor het personeel, anonieme redactie van wetenschappelijke artikels voorgesteld door de farmaceutische industrie (ghostwriting), diensten van het personeel als spreker bij congressen uitgevoerd met steun van de farmaceutische industrie, alsook de vraag van de faculteit om soortgelijke voorzieningen aan te moedigen op andere opleidingsplaatsen (stages op de medische praktijk of in het ziekenhuis). Tenslotte willen we ook achterhalen of er een comité werd opgericht dat nagaat of de besloten maatregelen worden uitgevoerd en of er sancties bestaan wanneer betrokken actoren deze reguleringen niet zouden toepassen. We zullen opnieuw contact met u opnemen na analyse van de gecommuniceerde informatie opdat u de mogelijkheid krijgt om de juistheid van onze resultaten en onze omschrijving van het beleid m.b.t. belangenconflicten van uw instelling te bevestigen.

Gezien we u enkel openbaar raadpleegbare documenten m.b.t. deze beleidsmaatregelen vragen blijft deze informatie niet vertrouwelijk en worden de faculteiten met naam genoemd. Vanzelfsprekend kunt u weigeren om met het project mee te werken en kunt u de samenwerking op elk moment stoppen. Openbaar beschikbare beleidsmaatregelen zullen daarentegen ook bij terugtrekking worden weerhouden.

Voor aanvullende informatie of inlichtingen kunt u ons steeds contacteren op bovenvermeld e-mailadres.

Wij danken u bij voorbaat voor de hulp die u ons kunt verlenen bij de realisatie van deze studie die belangrijk is voor de kwaliteit van de opleiding van toekomstige artsen en van patiëntenzorg en voor het beantwoorden van de verwachtingen van studenten zelf die betrokken zijn bij het thema belangenconflicten. Nu verscheidene schandalen de actualiteit haalden de afgelopen jaren moet worden vastgesteld dat deze thema’s m.b.t. onafhankelijkheid van het medisch beroep ten opzichte van de farmaceutische industrie meer dan ooit actueel zijn.

In afwachting van uw antwoord verblijven wij.

De verantwoordelijken van het onderzoek:

Elisabeth Bechet

Lucas Bechoux

Fabian Colle

Alizée Detiffe

Oriane De Vleeschouwer

Cécile Vanheuverzwijn

Florence Verhegghen

1. http://www.amsascorecard.org/ [↑](#footnote-ref-1)
2. Shnier A., Lexchin J., Mintzes B., Jutel A., Holloway K., (2013), Too Few, Too Weak: Conflict of Interest Policies at Canadian Medical Schools, PLoS ONE [↑](#footnote-ref-2)
3. Mason P., Tattersall M.H.N., (2011), Conflicts of interest : a review of institutional policy in Australian medical schools, The Medical Journal of Australia [↑](#footnote-ref-3)
4. Scheffer P, Guy-Coichard C, Outh-Gauer D, Calet-Froissart Z, Boursier M, Mintzes B, et al., (2017), Conflict of Interest Policies at French Medical Schools: Starting from the Bottom, PLoS ONE [↑](#footnote-ref-4)
5. Kesselheim A.S., (2013), Drug company gifts to medical students: the hidden curriculum, BMJ [↑](#footnote-ref-5)
6. King Marissa, Essick Connor, Bearman Peter, Ross Joseph S., (2013), Medical school gift restriction policies and physician prescribing of newly marketed psychotropic medications: difference-in-differences analysis, BMJ [↑](#footnote-ref-6)
7. Busing N., (2011), Canadian faculties of medicine not in denial, CMAJ [↑](#footnote-ref-7)
8. http://formindep.fr/charte-ethique-des-conferences-des-doyens-de-medecine-et-dodontologie/ [↑](#footnote-ref-8)
